# Chronic kidney disease related to *Loa loa* microfilaremia in a rural area of the Republic of Congo: a population-based cross-sectional study

**DOI:** 10.1186/s40249-025-01356-y

**Published:** 2025-08-21

**Authors:** Charlotte Boullé, Jérémy T. Campillo, Marlhand C. Hemilembolo, Elodie Lebredonchel, Valentin Dupasquier, Jean Claude Djontu, Sébastien D. S. Pion, Laurène Tardieu, Ludovic Rancé, François Missamou, Francine Ntoumi, Michel Boussinesq, Cédric B. Chesnais

**Affiliations:** 1 TransVIHMIINSERM U1175French National Research Institute for Sustainable Development (IRD) U233, Montpellier University, 911 Avenue Agropolis, 34394 Montpellier, France; 2 Department of Infectious and Tropical Diseases, Montpellier University Hospital, 39 Av. Charles Flahault, 34090 Montpellier, France; 3 National Onchocerciasis Control Programme (PNLO), Ministry of Health and Population, Brazzaville, Republic of Congo; 4 Department of Biochemistry, Greater Paris University Hospitals (AP-HP), Bichat, Paris, France; 5 Department of Cardiology, Montpellier University Hospital, Montpellier, France; 6 Congolese Foundation for Medical Research, Brazzaville, Republic of Congo; 7 Institute of Tropical Medicine, University of Tübingen, Tübingen, Germany; 8 Department of Nephrology, Montpellier University Hospital, Montpellier, France; 9 Department of Anesthesiology and Critical Care Medicine, Montpellier University Hospital, Montpellier, France

**Keywords:** Loiasis, Filariasis, Kidney, Ultrasonographic examination, Chronic kidney disease, Africa

## Abstract

**Background:**

Loiasis affects millions in Central Africa and, though historically considered benign, emerging data suggest possible renal involvement. This study investigated the association between *Loa* microfilaremia and renal function.

**Methods:**

We conducted a cross-sectional study in the Republic of Congo in May–June 2022. Renal function was assessed via estimated glomerular filtration rate (eGFR) using Chronic Kidney Disease—Epidemiology Collaboration (CKD-EPI) and European Kidney Function Consortium (EKFC) equations, and proteinuria and/or haematuria (renal abnormalities, RAb). Multinomial logistic regression assessed associations between microfilarial density (MFD) and chronic kidney disease (CKD), using EKFC with Dubois correction. Population attributable fractions were estimated from a logistic model including *Loa* microfilaremia as a binary variable (present *versus* absent).

**Results:**

Among 986 participants, CKD prevalence ranged from 13.4% [95% confidence interval (*CI*) 11.4–15.7%, CKD-EPI] to 17.6% (95% *CI* 15.3–20.1%, EKFC) for KDIGO stages 1–5, and from 3.0% (95% *CI* 2.1–4.3%, CKD-EPI) to 7.6% (95% *CI* 6.1–9.4%, EKFC) for stages 3–5. *Loa* MFD was associated with higher odds of CKD, particularly in individuals with RAb. Compared to amicrofilaremic participants, those with *Loa* MFD ≥ 20 000 mf/ml had significantly increased risk: adjusted relative risk ratio (aRRR) for CKD severity categories (≤ 2nd, 2nd–10th, 10th–50th, > 50th eGFR percentile) with RAb were 8.67 (95% *CI* 2.62–28.64, *P* = 0.021), 14.26 (95% *CI* 3.41–59.68, *P* < 0.001), 5.50 (95% *CI* 0.55–61.78, *P* = 0.145), and 26.21 (95% *CI* 1.64–417.84, *P* = 0.021). Population attributable fractions of CKD stages 1–5 to *Loa* microfilaremia was 14.7% (95% *CI* 4.3–24.0) and 30.1% (95% *CI* 16.2–42.8) for CKD stages 1–5 with RAb.

**Conclusions:**

This study provides the first epidemiological evidence linking loiasis to renal impairment, likely via glomerular damage. Given loiasis high endemicity in Central Africa, it may contribute to the burden of unexplained nephropathies. Longitudinal studies and renal biopsies are warranted to clarify underlying mechanisms.

**Graphical Abstract:**

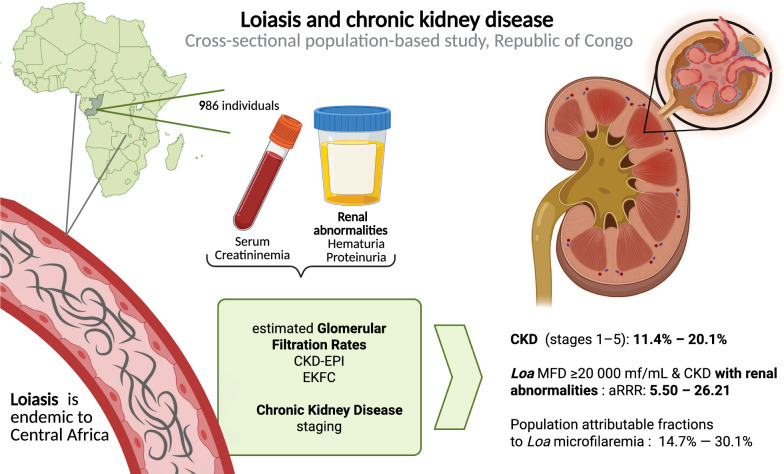

**Supplementary Information:**

The online version contains supplementary material available at 10.1186/s40249-025-01356-y.

## Background

Loiasis is a parasitic disease caused by *Loa loa*, a filarial worm transmitted from human to human by deerflies. About 15 million people are infected in Central Africa [1]. Adult parasites, which can live up to 20 years, release millions of larvae (microfilariae, mf) into the bloodstream, and the relatively stable microfilarial densities (MFD) over time can exceed 100,000 mf/ml in some individuals.

Loiasis is usually associated with marked eosinophilia, which may contribute to systemic complications, including coagulation and cardiac disorders [2, 3]. It is traditionally considered relatively benign, presenting with pruritus, subconjunctival migration of the adult worms, or transient angioedema (“Calabar swellings”), but recent results challenge this view. Retrospective cohort studies in Cameroon and the Republic of Congo have shown significantly reduced life expectancy in individuals with high *Loa* MFD, with mortality risk correlating with *Loa* MFD levels [4, 5].

Emerging data highlight the broader disease burden of loiasis, now implicated in cardiovascular and renal complications [6–8]. These findings may explain the excess mortality in heavily infected individuals and stress the need for further research on loiasis-associated morbidity, particularly renal involvement. Renal complications in loiasis have been reported for decades, mainly through case studies, ranging from isolated proteinuria to severe nephrotic syndrome and renal failure [9–17]. Renal biopsies in eight cases revealed membranous glomerulopathy, focal interstitial inflammation, or focal segmental glomerulosclerosis [11–13, 15, 16, 18, 19]. In some cases, renal function normalized after antifilarial treatment.

More recently, a strong association between *Loa* MFD and proteinuria severity was demonstrated, with a clear gradient effect as *Loa* MFD increased [7]. This was the first study, involving 990 individuals in the Republic of Congo, to confirm such a relationship, reinforcing decades of prior anecdotal observations. Building on these findings, we now assess the impact of *Loa* MFD on CKD in this population.

## Methods

### Study area and population

This cross-sectional study, nested within a cohort study, was conducted in May–June 2022 and included 990 participants aged 18 to 88 years living in 21 villages near Sibiti, Republic of Congo. Participants had been previously screened for *Loa* microfilaremia [20]. For the current study, individuals with blood microfilariae detected in 2021 were matched for sex and age (± 5 years) with two amicrofilaremic individuals from the same village. Detailed description of the study population has been published previously [8, 21].

The sample size for the underlying cohort study (*n* = 990) was calculated to provide 80% statistical power to detect an increased risk of malaria (1.4-fold) and pneumonia (2.4-fold) among microfilaremic compared to amicrofilaremic individuals, based on the hypothesis that loiasis may impair splenic function and thereby increase susceptibility to infections. However, due to the lack of prior data, the sample size was not calculated to evaluate differences in renal outcomes.

Participation in the study required informed consent from all individuals. The research was approved by the Ethics Committee of the Congolese Foundation for Medical Research (036/CIE/FCRM/2022) and by the National administrative authorities of Congo (376/MSP/CAB/UCPP-21).

### Sociodemographic and exposure covariables

Data on age, sex, anthropometric measurements (weight, height, and body mass index—BMI), and tobacco use were collected from each participant. Blood pressure was measured supine after 10 min rest. High blood pressure (HBP) was classified into three stages (see Table 1) and mean arterial pressure (MAP) was defined as $\frac{{{\text{Systolic BP + 2 }} \times {\text{Diastolic BP}}}}{{3}}$.

**Table 1:** Study population, serum creatinine levels and estimated glomerular filtration rate according to CKD-EPI or EKFC formulas, and Dubois correction for body surface area

	Individuals		Creatininemia (µmol/L)		eGFR (ml/min per 1.73m^2^)				eGFR (ml/min)			
					CKD-EPI		EKFC		CKD-EPI-Dubois		EKFC-Dubois	
	*N*	%	Mean	*SD*	Mean	*SD*	Mean	*SD*	Mean	*SD*	Mean	*SD*
Total (*N* = 986)*			71.2	19.2	98.6	19.2	89.1	1853	91.0	20.3	82.3	19.5
Age (years)												
18–28	86	8.7	69.4	16.2	122.2	14.5	104.8	12.2	116.2	15.8	99.7	13.8
29–38	137	13.9	69.6	15.2	113.7	13.7	104.2	12.9	106.2	14.7	97.3	13.8
39–48	191	19.4	70.8	20.7	105.3	14.8	99.5	14.6	98.6	15.2	93.2	14.8
49–58	252	25.6	70.8	15.8	95.8	13.7	87.5	13.6	89.4	14.4	81.7	14.2
59–68	204	20.7	70.5	15.7	87.4	12.7	77.0	12.3	78.7	13.6	69.4	13.2
> 68	116	11.8	77.5	30.9	78.3	17.0	67.2	15.1	67.2	15.6	57.7	13.9
Sex												
Female	368	37.3	60.8	14.2	95.9	19.2	85.0	18.8	83.6	19.4	74.1	18.6
Male	618	62.7	77.4	19.1	100.2	19.0	91.6	18.0	95.5	19.6	87.2	18.4
Proteinuria												
Negative	553	56.1	69.9	15.5	100.5	17.5	90.8	17.1	93.0	18.6	84.1	18.0
Traces	323	32.8	72.0	17.3	97.5	19.5	88.1	18.8	90.1	21.0	81.5	20.0
1 +	88	8.9	74.8	35.3	92.8	23.7	83.3	22.3	83.2	23.0	74.8	21.9
2 or 3 +	21	2.1	79.6	32.6	91.8	28.2	82.9	26.8	87.1	31.9	78.6	29.7
ACR > 3 mg/mmol												
No	937	95.0	71.0	17.7	99.0	18.8	89.4	18.2	91.5	19.9	82.7	19.1
Yes	49	5.0	75.1	37.5	91.7	24.5	82.3	23.8	82.9	26.0	74.6	25.2
Hematuria												
Negative	891	90.5	71.4	18.2	98.8	18.9	89.3	18.3	91.3	20.1	82.6	19.4
Traces	12	1.2	71.3	15.9	90.2	14.3	81.1	15.7	83.6	11.2	75.1	12.7
1 +	24	2.4	66.9	18.9	97.5	19.2	86.6	17.1	89.9	24.5	79.9	21.7
2 or 3 +	58	5.9	70.4	31.1	98.5	23.5	88.6	22.7	89.3	23.1	80.4	22.1
HBP												
No	636	64.6	70.7	18.3	103.2	18.4	93.7	17.3	95.6	19.8	86.8	18.6
Stage 1	203	20.6	71.4	20.6	93.7	17.7	84.8	17.8	85.5	19.0	77.5	19.0
Stage 2	87	8.8	72.3	18.1	87.4	16.4	77.3	16.6	80.8	17.0	71.6	17.1
Stage 3	59	6.0	75.1	24.3	82.5	17.0	72.4	15.7	75.8	17.5	66.6	16.4
Tobacco use												
No	796	81.4	70.4	19.7	97.8	19.4	88.1	18.6	90.2	20.3	81.3	19.4
Yes	182	18.6	74.9	16.9	101.8	18.1	93.5	17.8	94.4	20.1	86.8	19.6
PWV (≥ 12 m/sec)												
No	890	90.7	70.4	17.4	100.6	18.4	91.1	17.7	93.1	19.6	84.5	18.7
Yes	91	9.3	79.5	31.2	80.1	17.0	69.6	15.5	71.1	16.7	61.9	15.4
Lymphopenia ≤ 1200/µl												
No	966	98.0	71.4	19.3	98.5	19.2	89.0	18.6	91.0	20.4	82.3	19.6
Yes	20	2.0	61.6	12.9	101.9	18.1	91.3	18.0	92.0	18.2	82.7	18.7
Hypereosinophilia												
No	654	66.3	71.3	20.6	98.0	19.8	88.6	19.2	90.0	20.5	81.4	19.8
Yes	263	26.7	72.0	15.9	99.0	17.7	89.3	17.2	92.3	19.5	83.4	18.8
MD	69	7.0	67.3	16.3	103.3	18.2	93.1	16.8	96.1	20.9	86.7	19.4
*Loa* MFD (/ml of blood)												
0	633	64.2	71.9	19.9	97.6	19.4	88.1	18.7	90.3	20.3	81.5	19.5
1–7999	256	26.0	70.4	17.3	99.5	18.8	89.8	18.3	91.6	20.5	82.8	19.7
8000–19 999	65	6.6	67.8	20.5	104.6	17.3	95.7	16.8	96.1	18.8	88.0	18.0
≥ 20 000	32	3.2	72.4	16.8	99.1	19.1	90.5	18.7	91.4	21.7	83.6	20.7
*Ascaris lumbricoides*												
No	436	44.2	71.5	19.9	98.4	19.2	89.2	18.7	91.8	20.1	83.3	19.4
Yes	331	33.6	71.4	19.1	97.4	18.2	88.1	17.9	89.1	19.4	80.7	18.9
MD	219	22.2	70.3	17.8	100.8	20.5	90.4	19.1	92.4	22.1	82.8	20.6
*Trichuris trichuria*												
No	564	57.2	71.1	19.6	98.4	19.0	89.1	18.5	91.3	19.8	82.7	19.2
Yes	205	20.8	72.4	19.3	96.9	18.1	87.8	18.0	89.0	19.6	80.8	19.3
MD	217	22.0	70.4	17.9	100.7	20.5	90.2	19.2	92.3	22.2	82.7	20.6
Anti-*Plasmodium falciparum* IgG												
≤ 52 µg/ml	214	21.7	71.3	17.7	100.5	18.6	90.6	17.7	93.0	19.1	83.9	17.9
53–68 µg/ml	238	24.1	70.8	18.2	98.7	18.0	89.1	17.3	90.4	18.9	81.7	18.4
69–85 µg/ml	225	22.8	72.7	21.7	97.4	18.9	88.2	18.4	91.1	20.3	82.6	19.7
> 85 µg/ml	232	23.5	70.9	19.6	96.8	20.3	87.4	19.9	88.6	21.2	80.1	20.6
MD	77	7.8	69.0	17.3	102.3	21.2	92.7	20.1	94.8	24.2	86.0	22.5
Plasmodium smears												
Negative	970	98.4	71.3	19.3	98.5	19.2	89.1	18.6	91.0	20.3	82.3	19.5
Positive	16	1.6	66.6	8.4	104.1	16.8	91.7	16.6	94.1	21.4	83.0	20.3

^*^MD: missing data. 1 MD for HBP, 8 for tobacco, and 5 for PWV

*HBP* high blood pressure (No: Systolic BP (SBP) < 140 mmHg and Diastolic BP (DBP) < 90 mmHg, *stage 1* SBP ≥ 140 and < 160 and/or DBP ≥ 90 and < 100 mmHg, *stage 2* SBP ≥ 160 and < 180 and/or DBP ≥ 100 and < 110 mmHg, *stage 3* SBP ≥ 180 or DBP ≥ 110 mmHg), *PWV* pulse wave velocity, *eGFR* estimated glomerular filtration rate, *MFD* microfilarial density, *ACR* albumin- to creatinine ratio, *CKD-EPI* Chronic Kidney Disease—Epidemiology Collaboration, *EKFC* European Kidney Function Consortium

### Laboratory procedures for parasitic infections

Fifty µl of capillary blood were collected by finger-prick between 10 a.m. and 4 p.m. (to take into account the day and night fluctuation of *Loa* MFD) to prepare thick blood smears (TBS) which were then Giemsa-stained, and examined at 100 × magnification by experienced technicians to count *Loa* and *Mansonella perstans* mf. Each TBS was read twice; the arithmetic mean was recorded. Prior exposure to *Onchocerca volvulus* was assessed using the antibody detecting Onchocerciasis rapid test targeting Ov16 antigen (Drugs & Diagnostics for Tropical Diseases, San Diego, USA). Two skin snips were taken from seropositive individuals using a 2 mm Holth punch, incubated in saline at room temperature for 24 h and examined microscopically to count the emerged mf. *O. volvulus* MFD was calculated as the arithmetic mean of the two counts, expressed per snip.

*Schistosoma haematobium* infection was investigated in participants with haematuria detected by urine dipstick. Positive cases underwent urine filtration, Lugol staining, and microscopic examination for eggs. Soil-transmitted helminths (STH) were identified via microscopic examination of morning stool samples, transported within 6 h and processed immediately or after overnight storage at 6 °C. Stool smears were prepared using the Kato-Katz method and examined at 40 × magnification. For asymptomatic *Plasmodium* infection, thin blood films from venous blood (heparinized tube) were stained with RAL 555 (RAL Diagnostics, Martillac, France) and examined microscopically. An indirect ELISA was used to quantify the Immunoglobin G (IgG) antibodies levels against *P. falciparum* antigens [22].

### Biological examinations

Creatinine levels were measured for each patient in whole blood with a point-of-care device (iSTAT-1; Abbott Point of Care, Princeton, NJ, USA). For logistic reasons, glycated haemoglobin (Hb1Ac) and fasting serum total cholesterol level, triglycerides, HDL, and LDL were only measured in a random subset of patients, using a point-of-care device (Afinion 2, Abbott Rapid Diagnostics, Bièvres, France). Eosinophilia and lymphocyte counts were conducted using the HemoCue WBC DIFF system (HemoCue AB, Ängelholm, Sweden).

### Arterial stiffness assessment

Arterial stiffness was assessed through finger-toe pulse wave velocity (PWV) using the pOpmètre device (Axelife SAS, Paris, France), which records pulse waves via two infrared photodiode sensors at the finger and toe. This method is a validated, non-invasive alternative to carotid-femoral PWV, which requires skilled staff and femoral artery access [23].

### Outcome: chronic kidney disease

#### Ultrasound examination

The US examination of the kidneys was performed by experienced physicians (VD, LR) using a CX-50 device (Philips Medical Systems, Suresnes, France). Measurements included height, width, length. A second reading of the digital images (DICOM files) was performed to confirm and characterize kidney parenchymal or structure abnormalities (pyelocaliceal dilatation, cystic, or other description).

#### Urine collection and dipstick analysis

Each participant provided a morning midstream urine sample, following instructions from a trained nurse. Urinalysis was performed immediately using dipsticks (Reactif 10SL Urinalysis Strips, 41101-M, Nal von Minden, Moers, Germany) to detect proteinuria and haematuria. A single physician interpreted the results and did a second confirmatory dipstick if the initial result was positive. No discrepancies were observed. Haematuria was assessed as negative, traces, + , +  + , or +  +  + . Positive cases (≥ 1 +) underwent urine filtration and microscopic examination for *S. haematobium* eggs. Proteinuria was classified as negative, traces (< 0.3 g/l), level–1 (0.3–1 g/l), level–2 (1–3 g/l), or level–3 (> 3 g/l). Albumin-to-creatinine ratio (ACR, mg/mmol) was measured for proteinuria ≥ level–1 using the Afinion 2 device.

#### Estimated glomerular filtration rate (eGFR) and chronic kidney disease (CKD) classification

Given limited data on creatinine distribution and CKD in our population, we applied two formulas. First, the 2009 CKD-EPI equation without ethnicity factor, as current recommendation [24, 25]. Second, the EKFC equation, a newer formula more robust across all ages [26, 27]. Only one published Q-value (median normal creatininemia) exists for Central Africa [28], based on 494 urban individuals [25]. Therefore, we estimated our own Q-values by calculating median creatinine levels in individuals without HBP or renal abnormalities (proteinuria or haematuria). CKD-EPI and EKFC eGFRs were expressed in ml/min per 1.73 m^2^, and classified per KDIGO (Kidney Disease: Improving Global Outcomes Guidelines) categories [29]. Renal abnormalities (RAb) were defined as ACR > 3 mg/mmol or ≥ 1 + haematuria, excluding ultrasound-confirmed causes (9 cases of pyelocaliceal dilatation suggestive of lithiasis were excluded, as the associated haematuria was most likely of urological rather than nephrological origin and thus not indicative of intrinsic renal disease).

### Statistical analysis

Continuous variables with normal distribution were presented as mean ± standard deviation (*SD*); non-normal variables were reported as median (interquartile range). Explanatory variables included: age (continuous), sex (male/female), tobacco use (yes/no), MAP (continuous), PWV (≥ 12 vs. < 12 m/sec), lymphopenia (< 1200/µl), hypereosinophilia (> 1500/µl), *Ascaris lumbricoides* and/or *Trichuris trichiura* (present/absent), *Loa* MFD (0, 1–7999, 8000–19 999, ≥ 20 000 mf/ml), anti-*Plasmodium falciparum* IgG (quartiles), and *Plasmodium* smears (negative/positive). BMI was not included since anthropometric parameters are integrated into eGFR estimations. Lipid profiles and diabetes were not included due to substantial missing data (see Results). The PWV cutoff (12 m/sec) reflects its established association with organ damage [30]. HIV status was unknown, but lymphopenia < 1200/µl was used as a proxy.

In our study, 25% weighed < 50 kg (10% < 45 kg, range: 30–120 kg), 25% had a height < 157 cm (10% < 152 cm, range: 141–190 cm), and 25% had a BMI < 18.9 kg/m^2^ (10% < 17.8, range: 13.2–47.7). Given this heterogeneity, we chose to apply Dubois body surface area (BSA) correction to both CKD-EPI and EKFC equations to better assess associations. eGFR was calculated with CKD-EPI and then de-indexed using individual BSA via the Dubois formula: BSA = 0.007184 × Weight(kg)^0.425^ × Height(cm)^0.725^, resulting in CKD-Dubois and EKFC-Dubois values (ml/min).

First, a saturated linear regression model on eGFR was performed. Because KDIGO-CKD staging could not be used directly in the regression analysis—due to the necessity of adjusting for BSA and the small number of participants in advanced CKD stages, which limited statistical power—we defined three alternative severity thresholds based on the distribution of eGFR values in our study population to investigate a potential gradient effect of CKD severity. These were the median (Cutoff-1), the 10th percentile (Cutoff-2) – corresponding to the global prevalence of CKD –, and the 2nd percentile (Cutoff-3) –reflecting the prevalence of advanced CKD. Thresholds were calculated separately for each equation. Given that the presence of RAb is an essential component of CKD staging, it was incorporated in the categorization and thus the categories were: “ ≥ Cutoff-1 & no RAb”, “ ≥ Cutoff-1 & RAb”, “Cutoff-1 – Cutoff-2 & no RAb”, “Cutoff-1 – Cutoff-2 & RAb”, “Cutoff-2 – Cutoff-3 & no RAb”, “Cutoff-2 – Cutoff-3 & RAb”, “ < Cutoff-3 & no RAb”, “ < Cutoff-3 & RAb”.

Due to the ordered nature of outcome variables, ordinal logistic models were initially considered. However, they were rejected after a Brant test indicated a proportional odds violation. Instead, multinomial logistic regression was used. A manual stepwise backward selection (*P* < 0.100) was applied to the saturated model via the likelihood ratio test. To prevent convergence failure, *Loa* MFD was re-categorized into three groups (0, 1–19 999, ≥ 20 000 mf/ml). Finally, potential interactions between *Loa* MFD, age, sex, and eosinophilia were assessed using the likelihood ratio test. After finalizing the model, we extracted risk probabilities. We then estimated the population attributable fraction (PAF) from a logistic model including the final significant variables identified (age, sex, and *Loa* microfilaremia as a binary variable) using the KDIGO CKD classification with the EKFC formula.

All statistical analyses were performed using Stata 18 (StataCorps LP, College Station, Texas, USA).

## Results

### Description of the study population

Among 990 participants, 986 (618 men, 368 women) underwent creatinine testing (Table 1). *Loa* microfilaremia was found in 353/986 (35.8%), with a median MFD of 2440 mf/ml. No infection with hookworm, *S. mansoni* or *S. haematobium* was found (94 individuals with haematuria: 11 traces/ + ; 83 +  + / +  + +). *M. perstans* mf were found in 6/981 (0.6%; MFD range: 20–660 mf/ml). Ov16 RDT was positive in 22 (2.2%), but no *O. volvulus* mf was detected. HbA1c was measured in 238 participants; one had > 6.5%. Lipid profiles (*n* = 233) showed total cholesterol > 2 g/l in 231 (99.1%), LDL > 1.6 g/l in 150 (64.4%), HDL < 0.4 g/l in 1 (0.4%), and triglycerides > 1.5 g/l in 33 (13.0%).

### US kidney description

For the right kidney, mean (± standard deviation; range) dimensions were 9.36 cm (± 1.09; 2.72–14.60) in length, 3.39 cm (± 0.61; 1.74–9.78) in width, and 3.85 cm (± 0.56; 2.49–7.06) in thickness. For the left kidney, mean dimensions were 9.55 cm (± 1.13; 3.46–14.00), 3.45 cm (± 0.60; 1.30–5.45), and 3.95 cm (± 0.69; 2.09–11.04), respectively. Mean volumes were 65.49 cm^3^ (right) and 69.76 cm^3^ (± 24.06) (left). Nine individuals had a single kidney. A total of 109 kidney lesions were observed, predominantly cystic (none polycystic kidney disease), with 9 cases of pyelocaliceal dilatation.

### CKD classification

Table 2 summarizes the classification definitions. CKD prevalence was estimated at 13.4% [95% confidence interval (*CI*) 11.4–15.7%] with CKD-EPI and 17.6% (95% *CI* 15.3–20.1%) with EKFC. Although there may be no direct correspondence, using KDIGO thresholds, prevalence reached 16.6% (95% *CI* 14.4–19.1%) and 22.8% (95% *CI* 20.3–25.6%) for CKD-Dubois and EKFC-Dubois, respectively. CKD stage ≥ 3 prevalence was 3.0% (95% *CI* 2.1–4.3%) with CKD-EPI and 7.6% (95% *CI* 6.1–9.4%) with EKFC. Stage 4 included only 2 (CKD-EPI) and 3 (EKFC) individuals; no participants were classified as stage 5.

**Table 2:** KDIGO chronic kidney disease categories (with and without Dubois correction for body surface area) for CKD-EPI- and EKFC-based estimated glomerular filtration rates (eGFR)

	CKD-EPI-2009				EFKC			
	KDIGO		Dubois correction		KDIGO		Dubois correction	
Stages	*N* = 986	Percentage % (95% *CI*)	*N* = 986	Percentage % (95% *CI*)	*N* = 986	Percentage % (95% *CI*)	*N* = 986	Percentage % (95% *CI*)
CKD-EPI-2009								
Normal	853	86.6 (84.3–88.6)	821	83.4 (80.9–85.5)	812	82.4 (79.9–84.7)	821	77.2 (74.4–79.7)
Abnormal	133	13.4 (11.4–15.7)	165	16.6 (14.4–19.1)	174	17.6 (15.3–20.1)	211	22.8 (20.3–25.6)
CKD Stage 1	74	7.5 (6.2–9.3)	56	5.7 (4.4–7.3)	56	5.6 (4.3–7.2)	56	3.9 (2.9–5.4)
CKD Stage 2	28	2.8 (2.0–4.1)	39	4.0 (2.9–5.4)	43	4.5 (3.3–5.8)	39	4.7 (3.5–6.2)
CKD Stage 3a	20	2.0 (1.3–3.1)	55	5.6 (4.3–7.2)	61	6.3 (4.8–7.9)	55	11.0 (9.2–13.1)
CKD Stage 3b	8	0.8 (0.4–1.6)	9	0.9 (0.5–1.7)	11	1.1 (0.6–2.0)	9	2.6 (1.8–3.9)
CKD Stage 4	2	0.2 (0.05–0.8)	5	0.5 (0.2–1.2)	3	0.3 (0.01–1.0)	5	0.6 (0.3–1.4)
CKD Stage 5	0		0		0		0	

Stages definitions: Normal: eGFR ≥ 60 ml/min per 1.73 m^2^ and no RAb; CKD Stage 1: eGFR ≥ 90 ml/min per 1.73 m^2^ with Rab; CKD Stage 2: eGFR ≥ 60 and eGFR < 90 ml/min per 1.73 m^2^ and no Rab; CKD Stage 3a: eGFR ≥ 45 and eGFR < 60 ml/min per 1.73 m^2^; CKD Stage 3b: eGFR ≥ 30 and eGFR < 45 ml/min per 1.73 m^2^; CKD Stage 4: eGFR ≥ 15 and eGFR < 30 ml/min per 1.73 m^2^; CKD Stage 5: eGFR < 15 ml/min per 1.73 m^2^. Renal abnormalities (RAb) were defined as an albumin- to creatinine ratio > 3 mg/mmol or ≥ 1 cross of hematuria (excluding 9 cases due to pyelocaliceal dilation detected on renal ultrasound)

*CKD-EPI* Chronic Kidney Disease—Epidemiology Collaboration, *EKFC* European Kidney Function Consortium, *CI* confidence intervals, *KDIGO* Kidney Disease Improving Global Outcomes

### Factors influencing eGFR

Age was consistently associated with lower eGFR, regardless of RAb. For instance, in the EKFC-Dubois model, the coefficient for age was -0.85 (95% *CI* − 0.92, − 0.78; *P* < 0.001) in participants without RAb and -0.93 (95% *CI* − 1.16, − 0.70; *P* < 0.001) in those with RAb. Male sex was associated with higher eGFR only in participants without RAb (*β* = 8.54, 95% *CI* 6.60, 10.48; *P* < 0.001), but not in those with RAb (*β* = 5.08, 95% *CI*  − 1.58, 11.74; *P* = 0.133). Smoking was linked to lower eGFR, notably in those without RAb (*β* = − 2.16, 95% *CI* − 4.51, 0.18; *P* = 0.071 with EKFC-Dubois; *β* = − 3.41, 95% *CI* − 5.85, − 0.97; *P* = 0.006 with CKD-EPI-Dubois), but no significant association was found in those with RAb. PWV ≥ 12 m/sec was associated with lower eGFR in all subgroups. For instance, with EKFC-Dubois, in participants with RAb, *β* was − 10.68 (95% *CI* − 20.50, − 0.87; *P* = 0.033), and in participants without RAb, *β* was − 4.84 (95% *CI* − 8.20, − 1.47; P = 0.005). Lastly, loiasis showed a significant positive association: eGFR increased with *Loa* MFD, either as a categorical variable (Table 3) or as a continuous variable (Supplementary Table 1), but only in participants with Rab. For example, individuals with 8000–19 999 mf/ml had an increase in eGFR compared to amicrofilaremic individuals: *β* = 14.13 (95% *CI* 4.06, 24.20; *P* = 0.006 (EKFC-Dubois); those with ≥ 20 000 mf/ml also had higher eGFR, although not statistically significant (*β* = 9.01, 95% *CI* − 1.29, 19.31; *P* = 0.086).

**Table 3:** Multivariable linear regression on eGFR obtained with CKD-EPI or EKFC formula applying Dubois’s correction stratified on the presence or absence of renal abnormalities, incorporating *Loa* microfilarial density (MFD) as a categorical variable

	CKD-EPI Dubois				EKFC Dubois			
	Without RAb		With RAb		Without RAb		With RAb	
	*ß*-coef (95% *CI*)	*P*	*ß*-coef (95% *CI*)	*P*	*ß*-coef (95% *CI*)	*P*	ß-coef (95% *CI*)	*P*
Age (years)	− 0.92 (− 1.00, − 0.85)	< 0.001	− 0.98 (− 1.23, − 0.73)	< 0.001	− 0.85 (− 0.92, − 0.78)	< 0.001	− 0.93 (− 1.16, − 0.70)	< 0.001
Male	7.22 (5.21, 9.24)	< 0.001	4.56 (− 2.69, 11.81)	0.215	8.54 (6.60, 10.48)	< 0.001	5.08 (− 1.58, 11.74)	0.133
MAP	0.03 (− 0.03, 0.09)	0.337	0.03 (− 0.18, 0.24)	0.802	0.03 (− 0.03, 0.09)	0.307	0.02 (− 0.17, 0.21)	0.847
Tobacco use	− 3.41 (− 5.85, − 0.97)	0.006	− 0.73 (− 10.41, 8.95)	0.881	− 2.16 (− 4.51, 0.18)	0.071	0.80 (− 8.09, 9.69)	0.858
PWV	− 2.84 (− 6.33, 0.66)	0.112	− 10.69 (− 21.38, − 0.00)	0.050	− 4.84 (− 8.20, − 1.47)	0.005	− 10.68 (− 20.50, − 0.87)	0.033
Lymphopenia < 1200/µl	3.54 (− 3.25, 10.32)	0.306	12.13 (− 5.19, 29.46)	0.168	2.76 (− 3.76, 9.29)	0.406	13.21 (− 2.70, 29.12)	0.102
Hypereosinophilia	0.27 (− 0.14, 0.69)	0.195	1.65 (0.09, 3.20)	0.038	0.19 (− 0.21, 0.59)	0.355	1.65 (0.22, 3.07)	0.024
*Loa* MFD (Ref. 0 mf/ml)		0.390^a^		0.037^a^		0.259^a^		0.023^a^
1–7999 mf/ml	1.55 (− 0.59, 3.70)	0.155	1.55 (− 6.23, 9.32)	0.694	1.49 (− 0.57, 3.55)	0.157	1.04 (− 6.10, 8.18)	0.773
8000–19 999 mf/ml	2.09 (− 1.78, 5.96)	0.289	14.67 (3.70, 25.64)	0.009	2.99 (− 0.73, 6.71)	0.115	14.13 (4.06, 24.20)	0.006
> 20 000 mf/ml	− 0.98 (− 7.06, 5.10)	0.751	9.25 (− 1.97, 20.47)	0.105	− 0.43 (− 6.27, 5.42)	0.886	9.01 (− -1.29, 19.31)	0.086
*Ascaris lumbricoides*	− 0.20 (− 1.71, 1.32)	0.798	1.44 (− 5.37, 8.26)	0.675	0.08 (− 1.37, 1.54)	0.913	0.71 (− 5.55, 6.96)	0.822
*Trichuris trichuria*	0.13 (− 1.36, 1.63)	0.860	− 1.21 (− 7.93, 5.51)	0.721	− 0.22 (− 1.66, 1.21)	0.759	− 0.68 (− 6.85, 5.50)	0.828
Anti-*Plasmodium falciparum* IgG (Ref. ≤ 52 µg/ml)		0.091^a^		0.518^a^		0.051^a^		0.647^a^
53–68 µg/ml	0.48 (− 2.20, 3.16)	0.724	− 2.36 (− 12.75, 8.03)	0.653	0.51 (− 2.07, 3.09)	0.696	1.00 (− 8.54, 10.54)	0.835
69–85 µg/ml	1.81 (− 0.92, 4.54)	0.193	− 7.97 (− 18.09, 2.16)	0.121	1.94 (− 0.69, 4.56)	0.148	− 4.45 (− 13.74, 4.85)	0.345
> 85 µg/ml	2.32 (− 0.42, 5.07)	0.096	− 1.62 (− 11.21, 7.98)	0.739	2.31 (− 0.33, 4.95)	0.086	0.84 (− 7.97, 9.66)	0.849
*Plasmodium* smears	2.16 (− 5.07, 9.39)	0.557	− 11.32 (− 35.99, 13.36)	0.365	0.24 (− 6.71, 7.19)	0.946	− 17.01 (− 39.66, 5.65)	0.139

^a^Wald test

*coef* coefficient, *RAb* renal abnormalities, *MAP* mean arterial pressure, *PWV* pulse wave velocity, *eGFR* estimated glomerular filtration rate, *MFD* microfilarial density, *CKD-EPI* Chronic Kidney Disease—Epidemiology Collaboration, *EKFC* European Kidney Function Consortium

### Factors associated with CKD severity

Cutoff-1, cutoff-2 and cutoff-3 to categorize CKD severity corresponded to eGFRs of 90, 65, and 47 ml/min for the model using CKD-Dubois and 84, 56, and 41 ml/min for EKFC-Dubois. The repartition of the population in chronic kidney disease categories according to the study cutoffs using CKD-EPI- and EKFC-based estimated glomerular filtration rates is presented in Supplementary Table 2. The full models, including all tested variables, are presented in Supplementary Tables 3 and 4. In both models, only age, sex, smoking, and PWV were significantly associated with CKD severity (Table 4).

**Table 4:** Adjusted relative risk ratio of independently associated variables with severity categories of CKD in final multinomial models (using CKD-EPI with Dubois correction, upper panel, or EKFC with Dubois correction, lower panel)

	Age		Male sex		Tobacco use		PWV > 12 m/sec		*Loa* MFD 1–20 000 mf/ml		*Loa* MFD > 20 000 mf/ml	
	aRRR (95% *CI*)	*P*	aRRR (95% *CI*)	*P*	aRRR (95% *CI*)	*P*	aRRR (95% *CI*)	*P*	aRRR (95% *CI*)	*P*	aRRR (95% *CI*)	*P*
CKD-EPI Dubois correction (Ref. eGFR ≥ 90 & no RAb)												
eGFR [65–90[ & no RAb	1.13 (1.10–1.15)	< 0.001	0.34 (0.23–0.50)	< 0.001	1.44 (0.99–2.35)	0.143	1.08 (0.50–2.33)	0.845	0.91 (0.62–1.33)	0.611	1.09 (0.37–3.24)	0.884
eGFR [47–65[ & no RAb	1.21 (1.17–1.25)	< 0.001	0.18 (0.09–0.33)	< 0.001	3.27 (1.45–7.36)	0.004	1.05 (0.41–2.71)	0.916	0.66 (0.34–1.29)	0.229	1.44 (0.25–8.21)	0.678
eGFR < 47 & no RAb	1.28 (1.20–1.37)	< 0.001	0.14 (0.04–0.45)	0.001	5.08 (1.16–22.24)	0.031	1.67 (0.42–6.60)	0.463	0.30 (0.06–1.44)	0.130	NA	–
eGFR ≥ 90 & RAb	1.01 (0.98–1.03)	0.929	0.35 (0.18–0.67)	0.002	1.69 (0.82–3.48)	0.156	2.70 (0.65–11.20)	0.171	1.91 (1.04–3.49)	0.036	8.08 (2.67–24.41)	< 0.001
eGFR [65–90[ & RAb	1.19 (1.14–1.24)	< 0.001	0.09 (0.04–0.21)	< 0.001	1.22 (0.32–4.59)	0.773	1.57 (0.25–2.18)	0.412	3.60 (1.58–8.18)	0.002	14.85 (3.19–69.05)	0.001
eGFR [47–65[ & RAb	1.17 (1.09–1.25)	< 0.001	0.30 (0.10–1.00)	0.050	0.83 (1.00–7.01)	0.861	4.03 (0.96–16.87)	0.057	2.10 (0.63–6.03)	0.228	6.35 (0.57–70.20)	0.132
eGFR < 47 & RAb	1.15 (1.04–1.28)	0.007	0.33 (0.05–2.30)	0.265	2.81 (0.26–30.94)	0.399	3.94 (0.41–38.21)	0.237	0.61 (0.06–6.28)	0.681	11.38 (0.91–143.80)	0.060
EKFC Dubois correction (Ref. eGFR ≥ 84 & no RAb)												
eGFR [56–84[ & no RAb	1.12 (1.10–1.14)	< 0.001	0.30 (0.20–0.45)	< 0.001	1.49 (0.92–2.40)	0.102	2.53 (0.92–6.97)	0.102	0.96 (0.66–1.40)	0.817	1.21 (0.40–3.54)	0.817
eGFR [41–56[ & no RAb	1.26 (1.21–1.30)	< 0.001	0.10 (0.05–0.21)	< 0.001	4.33 (1.77–10.56)	0.001	2.22 (0.69–7.17)	0.184	0.40 (0.16–1.17)	0.105	1.64 (0.27–9.99)	0.591
eGFR < 41 & no RAb	1.34 (1.24–1.45)	< 0.001	0.12 (0.04–0.45)	0.002	7.89 (1.69–36.89)	0.009	2.85 (56–14.44)	0.206	0.30 (0.06–1.54)	0.150	NA	–
eGFR ≥ 84 & RAb	0.99 (0.97–1.02)	0.578	0.29 (0.14–0.59)	0.001	1.66 (0.76–3.62)	0.205	2.48 (0.26–23.51)	0.429	2.14 (1.12–4.08)	0.021	8.67 (2.62–28.64)	0.021
eGFR [56–84[ & RAb	1.19 (1.14–1.23)	< 0.001	0.10 (0.05–0.23)	< 0.001	1.79 (0.60–5.39)	0.299	1.66 (0.42–6.52)	0.467	2.68 (1.25–5.72)	0.011	14.26 (3.41–59.68)	< 0.001
eGFR [41–56[ & RAb	1.18 (1.10–1.26)	< 0.001	0.28 (0.09–0.90)	0.033	1.88 (0.37–9.67)	0.450	7.51 (1.60–35.39)	0.011	1.60 (0.49–5.17)	0.436	5.50 (0.55–61.78)	0.145
eGFR < 41 & RAb	1.17 (1.03–1.32)	0.017	0.20 (0.02–1.64)	0.132	NA	–	11.38 (1.85–152.70)	0.046	1.10 (0.10–12.73)	0.939	26.21 (1.64–417.84)	0.021

*RAb* renal abnormalities, *eGFR* estimated glomerular filtration rate, *CKD-EPI* Chronic Kidney Disease—Epidemiology Collaboration, *EKFC* European Kidney Function Consortium, *CI* confidence intervals, *aRRR* adjusted relative risk ratio, *MFD* microfilarial density, *NA* Not applicable. *Loa* MFD variable aRRR compared to amicrofilaremic individuals

Age showed no association in early CKD but was progressively linked to increasing severity. For example, in the CKD-EPI Dubois model, the aRRR per year increase in age was 1.19 (95% *CI* 1.14–1.24, *P* < 0.001) for the category eGFR 65–90 ml/min with RAb, and 1.28 (95% *CI* 1.20–1.37, *P* < 0.001) for eGFR < 47 ml/min without RAb. Male sex was protective (e.g. aRRR = 0.09, 95% *CI* 0.04–0.21, *P* < 0.001 for eGFR 65–90 with RAb, and 0.14, 95% *CI* 0.04–0.45, *P* = 0.001 for eGFR < 47 without RAb). Smoking tended to increase risk, especially in stages without RAb (aRRR = 1.49, 95% *CI* 0.92–2.40, *P* = 0.101; aRRR = 4.33, 95% *CI* 1.77–10.56, *P* = 0.001, and aRRR = 7.89, 95% *CI* 1.69–36.89, *P* = 0.009; for eGFR EKFC Dubois of [56–84, 41–56], and < 41 ml/min without RAb, respectively). High PWV affected all stages, with particularly elevated values in advanced stages with RAb (for instance, aRRR = 11.38, 95% *CI* 1.85–152.70, *P* = 0.046, for eGFR < 41 ml/min with RAb according to EKFC Dubois). Higher *Loa* MFD was significantly associated with CKD compared to amicrofilaremic individuals, showing a dose–response relationship regardless of CKD category, especially in those with RAb. Compared to amicrofilaremic individuals, those with *Loa* MFD between 1 and 20 000 mf/ml had aRRR of 2.14 (95% *CI* 1.12–4.08, *P* = 0.021) for the eGFR ≥ 84 ml/min category (EKFC Dubois) with RAb, 2.68 (95% *CI* 1.25–5.72, *P* = 0.011) for 56–84 ml/min, 1.60 (95% *CI* 0.49–5.17, *P* = 0.436) for 41–56 ml/min, and 1.10 (95% *CI* 0.10–12.73, *P* = 0.939) for < 41 ml/min. Similarly, individuals with > 20 000 mf/ml had aRRR of 8.67 (95% *CI* 2.62–28.64, *P* = 0.021) for eGFR ≥ 84 ml/min, 14.26 (95% *CI* 3.41–59.68, *P* < 0.001) for 56–84 ml/min, 5.50 (95% *CI* 0.55–61.78, *P* = 0.145) for 41–56 ml/min, and 26.21 (95% *CI* 1.64–417.84, *P* = 0.021) for < 41 ml/min. However, no clear linear trend was seen regarding CKD severity; all groups with RAb exhibited consistent risks across *Loa* MFD levels.

Table 5 shows predicted CKD risk by *Loa* MFD (EKFC-Dubois formula). No significant interactions were found between *Loa* MFD and sex or hypereosinophilia (*P* = 0.191, and 0.352, respectively), but a significant interaction was observed with age (*P* < 0.001), especially in individuals with RAb. For clarity, this interaction is illustrated graphically (Supplementary Fig. 1). In participants < 50 years, higher *Loa* MFD quadrupled the probability of classifying as normal with RAb. In those ≥ 50 years, *Loa* MFD increased risk of more advanced CKD stages with RAb.

**Table 5:** Predicted probabilities and 95% confidence interval for belonging to each severity category of CKD derived from EKFC with Dubois correction, depending on *Loa* MFD

	*Loa* MFD 0 mf/ml	*Loa* MFD 1–20 000 mf/ml	*Loa* MFD > 20 000 mf/ml
eGFR ≥ 84 & no RAb	45.6 (42.5–48.6)	43.6 (39.2–48.0)	29.5 (16.1–42.9)
eGFR [56–84[ no RAb	36.6 (33.2–40.0)	35.6 (30.8–40.4)	23.3 (9.6–37.0)
eGFR [41–56[ & no RAb	8.9 (6.9–10.9)	4.1 (2.0–6.1)	6.7 (2.8–14.9)
eGFR < 41 & no RAb	1.6 (0.7–2.6]	0.6 (0.2–1.4)	NA
eGFR ≥ 84 & RAb	3.4 (2.0–4.8)	6.9 (4.1–9.7)	17.7 (5.0–30.4)
eGFR [56–84[ & RAb	2.5 (1.3–3.7)	7.0 (4.2–9.8)	16.3 (4.3–28.3)
eGFR [41–56[ & RAb	1.1 (0.3–1.9)	1.9 (0.4–3.4)	3.1 (0.5–8.9)
eGFR < 41 & RAb	0.3 (0.01–0.7)	0.3 (0.1–1.0)	3.5 (0.8–10.2)

*RAb* renal abnormalities, *eGFR* estimated glomerular filtration rate, *EKFC* European Kidney Function Consortium, *MFD* microfilarial density, *NA* Not applicable

The PAF of CKD to female sex was the highest, estimated at 22.3% (95% *CI* 10.2–32.7%) for CKD ≥ 1, and 23.2% (95% *CI* 7.9–35.9%) for CKD ≥ 1 with RAb. This was followed by the PAF of *Loa* microfilaremia, estimated at 14.7% (95% *CI* 4.3–24.0%) of CKD cases, and up to 30.1% (95% *CI* 16.2–42.8%) with RAb. Arterial stiffness accounted for 4.4% (95% *CI* 0.0–10.1%) and 3.7% (95% *CI* 0.0–10.2%), respectively, while tobacco use explained 3.5% (95% *CI* 0.0–8.9%) and 2.8% (95% *CI* 0.0–10.4%) (Supplementary Table 5).

To clarify the lack of association with MAP, post-hoc analysis showed a significant interaction with age (*P* = 0.001): MAP affected individuals < 50 years but not older participants.

## Discussion

This study is the first to highlight a relationship between *Loa* microfilaremia and CKD severity. We demonstrate for the first time a dose–response relationship between *Loa* MFD and CKD, though without a clear gradient with CKD severity. Interestingly, eGFR increases with parasitic density. This finding should be interpreted in light of the observation that, among individuals ≤ 50 years, higher *Loa* MFD raises the probability of early CKD stages, whereas among those > 50, higher *Loa* MFD increased probability of being in more advanced stages. This supports the hypothesis that chronic microfilaremia exposure may be required to develop renal impairment, possibly preceded by glomerular hyperfiltration, similar to diabetes. The association’s specificity to CKD categories with renal abnormalities suggests preferential glomerular involvement.

Our results align with existing literature. As widely described, age is a key determinant of CKD. Women were more at risk, independently of other factors, consistent with elsewhere [31]. Tobacco use was strongly associated with CKD, independently of MAP and PWV. The absence of association with MAP may seem surprising, but disappeared after age adjustment, likely due to an age-MAP interaction. This suggests that in younger individuals (< 50 years), blood pressure influences CKD risk, whereas age dominates in older participants.

Arterial stiffness, well established as a CKD factor [32], showed a stronger association in categories with renal abnormalities, consistent with its mechanism: increased pulsatile pressure damaging renal microcirculation, leading to hyperfiltration and proteinuria. Although the effect of arterial stiffness was largely independent of blood pressure, inclusion of the latter in the model led to ~ 20% reduction in arterial stiffness coefficients, suggesting partial mediation via hypertension-related nephroangiosclerosis.

We observed no impact of arterial stiffness or MAP on the *Loa*-CKD association, arguing against a primarily circulatory mechanism. However, we cannot fully exclude it, as previous studies linked *Loa* MFD to arterial stiffness [8]. Available case reports suggest glomerular involvement to be the predominant form of kidney damage, in the form of membranoproliferative glomerulonephritis linked to complement activation, membranous nephropathy with IgG, complement, and/or immune complex deposition [33–36], or even renal amyloidosis [37, 38] due to chronic low-grade inflammation. Tubulointerstitial involvement appears less likely but has been reported [19], particularly post-antifilarial treatment [39]. Nonetheless, we cannot entirely exclude a secondary inflammatory effect related to vascular stiffness that could contribute to kidney damage. Only kidney biopsy could definitively characterize the type and mechanism of renal involvement.

We also provide figures of CKD prevalence in the general population of the Republic of Congo, estimated between 13.4% (95% *CI* 11.4–15.7%, CKD-EPI) and 17.6% (95% *CI* 15.3–20.1%, EKFC) for stages 1–5, and between 3.0% (95% *CI* 2.1–4.3%, CKD-EPI) and 7.6% (95% *CI* 6.1–9.4%, EKFC) for stages 3–5. Currently, CKD prevalence is estimated at 13.4% (95% *CI* 11.7–15.1) for CKD ≥ 1 and 10.6% (95% *CI* 9.2–12.2) for CKD stages ≥ 3, with estimates for Africa at 8.7% (95% *CI* 1.3–16.0) for CKD ≥ 1 and 7.6% (95% *CI* 6.1–9.1) for CKD stages ≥ 3 [40]. A second meta-analysis focusing on African studies reported an overall CKD prevalence of 10.1% (95% *CI* 9.8–10.5) [41]. Central African countries seem to have higher CKD prevalence, as in South Kivu (12.2%, 95% *CI* 10.2–14.2) [42], or rural Cameroon (3–14.1%) [43]; our estimates fall within the upper range of these figures. We also show that 14.7% (95% *CI* 4.3–24.0%) of CKD cases could have been avoided if no individuals had been microfilaremic. Together, our results suggest that loiasis may partly explain CKD cases of unknown cause, for instance in Cameroon, where the aetiology of CKD has been reported to be unknown in 13.5–17.0% of cases [43].

Our study has several limitations. First, CKD diagnosis relied on a single creatinine measurement, which does not strictly meet the criteria for CKD classification. HIV status was unavailable; however, no lymphopenia were observed in CKD stage ≥ 3, limiting likely HIV impact. Data on tenofovir exposure, which could contribute to tubulopathies, were also lacking. Given population heterogeneity and frequent short stature, true CKD prevalence likely falls within a range around our estimates, hence our application of Dubois corrections. Cystatin C measurement would have improved classification. Lastly, our study confirms the absence of association between STH, and malaria on kidney function in this population.

## Conclusions

We demonstrate for the first time the detrimental impact of loiasis on kidney function, likely mediated by glomerular involvement after chronic exposure. Given its high prevalence in Central Africa, loiasis may contribute significantly to unexplained nephropathies at the population level.

## Supplementary Information

Additional file 1.

Additional file 2.

## Data Availability

Anonymized data will be hosted on the IRD dataverse server: 10.23708/FUWEBT and its terms of use will be those in force on the hosting site.

## Abbreviations

ACR: Albumin to creatinine ratio
aRRR: Adjusted relative risk ratio
BMI: Body mass index
BSA: Body surface area
*CI*: Confidence interval
CKD: Chronic kidney disease
CKD-EPI: Chronic kidney disease epidemiology collaboration
Coef: Coefficient
DBP: Diastolic blood pressure
eGFR: Estimated glomerular filtration rate
EKFC: European kidney function consortium
HBP: High blood pressure
HIV: Human immunodefficiency virus
KDIGO: Kidney disease improving global outcomes
MAP: Mean arterial pressure
mf: Microfilariae
MFD: Microfilarial density
PAF: Population attributable fraction
PWV: Pulse wave velocity
RAb: Renal abnormalities
SBP: Systolic blood pressure
STH: Soil-transmitted helminths
TBS: Thick blood smears
