# Supplementary material for: Chronic kidney disease related to Loa loa microfilaremia in a rural area of the Republic of Congo: a population-based cross-sectional study
Source: Infect Dis Poverty. 2025 Aug 21;14:88. doi: 10.1186/s40249-025-01356-y (PMC12369049; doi:10.1186/s40249-025-01356-y)
Supplement: Supplementary file 1 — Additional file 1. [file 40249_2025_1356_MOESM1_ESM.docx]

**Supplementary Material: Chronic kidney disease related to *Loa loa* microfilaremia in a rural area of the Republic of Congo: a population-based cross-sectional study**

**Supplementary Table 1. Multivariable linear regression on eGFR obtained with CKD-EPI or EKFC formula applying Dubois’s correction stratified on the presence or absence of renal abnormalities, incorporating *Loa* microfilarial density (MFD) as a continuous variable**

|  | CKD-EPI Dubois | | | | EKFC Dubois | | | |
| --- | --- | --- | --- | --- | --- | --- | --- | --- |
|  | Without RAb | | With RAb | | Without RAb | | With RAb | |
|  | ß-coef [95% *CI*] | *P* | ß-coef [95% *CI*] | *P* | ß-coef [95% *CI*] | *P* | ß-coef [95% *CI*] | *P* |
| Age (years) | -0.92 [-1.00, -0.85] | <0.001 | -0.99 [-1.24, -0.73] | <0.001 | -0.85 [-0.92, -0.78] | <0.001 | -0.94 [-1.17, -0.71] | <0.001 |
| Male | 7.32 [5.31, 9.33] | <0.001 | 5.42 [-1.91, 12.75] | 0.145 | 8.63 [6.70, 10.56] | <0.001 | 5.90 [-0.85, 12.65] | 0.086 |
| MAP | 0.03 [-0.03, 0.09] | 0.367 | 0.04 [-0.17, 0.25] | 0.724 | 0.03 [-0.03, 0.09] | 0.338 | 0.03 [-0.17, 0.22] | 0.783 |
| Tobacco use | -3.40 [-5.84, -0.97] | 0.006 | -0.30 [-9.79, 9.19] | 0.950 | -2.12 [-4.46, 0.22] | 0.076 | 1.08 [-7.66, 9.82] | 0.807 |
| PWV | -2.74 [-6.24, 0.75] | 0.124 | -10.23 [-21.08, 0.61] | 0.064 | -4.74 [-8.11, -1.38] | 0.006 | -10.19 [-20.18, -0.20] | 0.046 |
| Lymphopenia <1200/µL | 3.59 [-3.20, 10.37] | 0.300 | 14.12 [-3.39, 31.64] | 0.113 | 2.80 [-3.72, 9.33] | 0.399 | 15.14 [-0.99, 31.26] | 0.066 |
| Hypereosinophilia | 0.26 [-0.15, 0.67] | 0.217 | 1.65 [0.08, 3.23] | 0.040 | 0.18 [-0.22, 0.57] | 0.386 | 1.65 [0.20, 3.10] | 0.026 |
| *Loa* MFD (mf/mL) | 0.00 [-0.00, 0.00] | 0.516 | 0.00 [-0.00, 0.00]^a^ | 0.062 | 0.00 [-0.00, 0.00] | 0.357 | 0.00 [0.00, 0.00]^b^ | 0.044 |
| *Ascaris lumbricoides* | -0.18 [-1.70, 1.33] | 0.813 | 1.47 [-5.41, 8.35] | 0.672 | 0.09 [-1.37, 1.54] | 0.908 | 0.69 [-5.64, 7.02] | 0.829 |
| *Trichuris trichuria* | 0.11 [-1.38, 1.61] | 0.881 | -1.31 [-8.10, 5.49] | 0.703 | -0.23 [-1.67, 1.20] | 0.748 | -0.73 [-6.98, 5.52] | 0.817 |
| Anti-*Plasmodium falciparum* IgG (Ref. ≤52 µg/mL) |  | 0.085^c^ |  | 0.632^c^ |  | 0.048^c^ |  | 0.735^c^ |
| 53–68 µg/mL | 0.47 [-2.21, 3.15] | 0.733 | -1.41 [-11.90, 9.08] | 0.790 | 0.49 [-2.09, 3.07] | 0.710 | 1.98 [-7.68, 11.64] | 0.685 |
| 69–85 µg/mL | 1.87 [-0.86, 4.59] | 0.180 | -7.12 [-17.37, 3.14] | 0.171 | 2.00 [-0.63, 4.62] | 0.135 | -3.59 [-13.04, 5.85] | 0.452 |
| >85 µg/mL | 2.36 [-0.38, 5.11] | 0.091 | -1.93 [-11.68, 7.82] | 0.695 | 2.35 [-0.29, 4.98] | 0.081 | 0.53 [-8.45, 9.51] | 0.906 |
| *Plasmodium* smears | 2.34 [-4.89, 9.57] | 0.525 | -6.80 [-31.38, 17.79] | 0.584 | 0.40 [-6.56, 7.35] | 0.910 | -12.80 [-35.44, 9.84] | 0.264 |

^a^ ß-coef [95% CI] = 0.000248 [-0.000014–0.0004821]. ^b^ ß-coef [95% CI] = 0.00002378 [0.0000221–0.0004534]; ^c^ Wald test. Abbreviations: RAb: renal abnormalities; MAP: mean arterial pressure; PWV: pulse wave velocity; eGFR: estimated glomerular filtration rate; MFD: microfilarial density; ACR: albumin- to creatinine ratio; CKD-EPI: Chronic Kidney Disease - Epidemiology Collaboration; EKFC: European Kidney Function Consortium.

**Supplementary Table 2. Repartition of the population in chronic kidney disease categories according to the study cutoffs using CKD-EPI- and EKFC-based estimated glomerular filtration rates.**

|  | CKD-EPI 2009 | | | EKFC | | |
| --- | --- | --- | --- | --- | --- | --- |
| Category | N | % | [95% *CI*] | N | % | [95% *CI*] |
| Normal | 477 | 48.4% | [45.3–51.6] | 438 | 44.5% | [41.4–47.6] |
| Normal with RAb | 56 | 5.7% | [4.4–7.3] | 49 | 5.0% | [3.8–6.5] |
| Mild without RAb | 312 | 31.7% | [28.8–34.7] | 354 | 35.9% | [33.0–39.0] |
| Mild with RAb | 35 | 3.6% | [2.6–4.9] | 42 | 4.3% | [3.2–5.7] |
| Moderate without RAb | 73 | 7.4% | [5.9–9.2] | 72 | 7.3% | [5.8–9.1] |
| Moderate with RAb | 13 | 1.3% | [0.8–2.3] | 14 | 1.4% | [0.8–2.4] |
| Advanced without RAb | 14 | 1.4% | [0.8–2.4] | 12 | 1.2% | [0.7–2.1] |
| Advanced with RAb | 5 | 0.5% | [0.2–1.2] | 4 | 0.4% | [0.2–1.1] |

Category definitions: CKD-EPI 2009: Normal: eGFR ≥90 (with or without RAb); Mild: eGFR 65–89 (with or without RAb); moderate: eGFR 47–64(with or without RAb); advanced eGFR <47 (with or without RAb). EKFC: Normal: eGFR ≥84 (with or without RAb); Mild: eGFR 56–83 (with or without RAb); moderate: eGFR 41–55 (with or without RAb); advanced eGFR <41 (with or without RAb). Abbreviations: CKD-EPI: Chronic Kidney Disease - Epidemiology Collaboration; EKFC: European Kidney Function Consortium; CI: confidence intervals; RAb: renal abnormalities.

**Supplementary Table 3. Adjusted relative risk ratio of independently associated variables with severity categories of CKD in saturated multinomial model using CKD-EPI with Dubois correction.**

| **Ref. eGFR ≥90 & no Ab** | **Adjusted RRR** | **Low 95%*CI*** | **High 95%*CI*** | ***P*** |
| --- | --- | --- | --- | --- |
| **eGFR ≥90 & Ab** |  |  |  |  |
| Age (y.o.) | 0.99 | 0.96 | 1.01 | 0.573 |
| Male | 0.38 | 0.19 | 0.75 | 0.005 |
| MBP | 1.00 | 0.98 | 1.02 | 0.839 |
| Tabacco | 1.55 | 0.73 | 3.31 | 0.252 |
| PWV | 3.15 | 0.72 | 13.81 | 0.128 |
| Lymphopenia | 3.76 | 0.85 | 16.60 | 0.081 |
| Hypereosinophilia | 1.05 | 0.94 | 1.18 | 0.373 |
| *Loa* MFD (Ref. 0 mf/mL) |  |  |  |  |
| 1-19.999mf/mL | 1.98 | 1.06 | 3.69 | 0.031 |
| >20.000 mf/mL | 8.76 | 2.75 | 27.97 | <0.001 |
| Ascaris | 0.88 | 0.52 | 1.48 | 0.629 |
| Trichuris | 0.98 | 0.58 | 1.63 | 0.927 |
| IgG-Plasmodium (Ref. ≤52 µg/mL) |  |  |  |  |
| 53-68 µg/mL | 0.83 | 0.36 | 1.92 | 0.666 |
| 69-85 µg/mL | 0.58 | 0.24 | 1.48 | 0.260 |
| >85 µg/mL | 0.92 | 0.39 | 2.17 | 0.857 |
| MD | 1.24 | 0.44 | 3.51 | 0.688 |
| *Plasmodium* smears | 0.89 | 0.11 | 7.88 | 0.921 |
| **eGFR ≥65 & eGFR <90 & no Ab** | |  |  |  |
| Age (y.o.) | 1.13 | 1.11 | 1.15 | <0.001 |
| Male | 0.34 | 0.22 | 0.50 | <0.001 |
| MBP | 0.99 | 0.98 | 1.00 | 0.408 |
| Tabacco | 1.42 | 0.87 | 2.34 | 0.161 |
| PWV | 1.06 | 0.48 | 2.25 | 0.880 |
| Lymphopenia | 0.70 | 0.19 | 2.55 | 0.587 |
| Hypereosinophilia | 0.93 | 0.85 | 1.01 | 0.077 |
| *Loa* MFD (Ref. 0 mf/mL) |  |  |  |  |
| 1-19.999mf/mL | 0.87 | 0.59 | 1.28 | 0.472 |
| >20.000 mf/mL | 1.08 | 0.35 | 3.35 | 0.888 |
| Ascaris | 1.22 | 0.94 | 1.59 | 0.132 |
| Trichuris | 0.86 | 0.66 | 1.11 | 0.240 |
| IgG-Plasmodium (Ref. ≤52 µg/mL) |  |  |  |  |
| 53-68 µg/mL | 0.98 | 0.58 | 1.66 | 0.959 |
| 69-85 µg/mL | 1.14 | 0.66 | 1.96 | 0.629 |
| >85 µg/mL | 0.87 | 0.51 | 1.49 | 0.622 |
| MD | 0.60 | 0.26 | 1.37 | 0.227 |
| *Plasmodium* smears | 0.83 | 0.17 | 3.92 | 0.811 |
| **eGFR ≥65 & eGFR <90 & Ab** | |  |  |  |
| Age (y.o.) | 1.19 | 1.14 | 1.24 | <0.001 |
| Male | 0.08 | 0.03 | 0.21 | <0.001 |
| MBP | 0.99 | 0.97 | 1.02 | 0.754 |
| Tabacco | 1.18 | 0.31 | 4.57 | 0.808 |
| PWV | 0.54 | 0.14 | 2.14 | 0.381 |
| Lymphopenia | 0.70 | 0.06 | 8.23 | 0.775 |
| Hypereosinophilia | 0.86 | 0.67 | 1.10 | 0.237 |
| *Loa* MFD (Ref. 0 mf/mL) |  |  |  |  |
| 1-19.999mf/mL | 3.35 | 1.45 | 7.71 | 0.004 |
| >20.000 mf/mL | 14.62 | 3.02 | 70.74 | 0.001 |
| Ascaris | 0.92 | 0.43 | 1.95 | 0.826 |
| Trichuris | 1.19 | 0.57 | 2.50 | 0.642 |
| IgG-Plasmodium (Ref. ≤52 µg/mL) |  |  |  |  |
| 53-68 µg/mL | 0.63 | 0.19 | 2.14 | 0.466 |
| 69-85 µg/mL | 1.12 | 0.35 | 3.57 | 0.850 |
| >85 µg/mL | 0.82 | 0.26 | 2.58 | 0.732 |
| MD | 0.95 | 0.16 | 5.54 | 0.957 |
| *Plasmodium* smears | 0.88 | 0.07 | 12.00 | 0.926 |
| **eGFR ≥47 & eGFR <65 & no Ab** | |  |  |  |
| Age (y.o.) | 1.22 | 1.17 | 1.27 | <0.001 |
| Male | 0.17 | 0.09 | 0.32 | <0.001 |
| MBP | 1.00 | 0.98 | 1.02 | 0.688 |
| Tabacco | 3.41 | 1.49 | 7.80 | 0.004 |
| PWV | 1.01 | 0.38 | 2.68 | 0.978 |
| Lymphopenia | 0.59 | 0.07 | 4.62 | 0.612 |
| Hypereosinophilia | 0.93 | 0.80 | 1.08 | 0.362 |
| *Loa* MFD (Ref. 0 mf/mL) |  |  |  |  |
| 1-19.999mf/mL | 0.64 | 0.32 | 1.27 | 0.203 |
| >20.000 mf/mL | 1.56 | 0.27 | 9.04 | 0.618 |
| Ascaris | 0.97 | 0.55 | 1.67 | 0.891 |
| Trichuris | 1.03 | 0.59 | 1.78 | 0.915 |
| IgG-Plasmodium (Ref. ≤52 µg/mL) |  |  |  |  |
| 53-68 µg/mL | 0.71 | 0.29 | 1.70 | 0.440 |
| 69-85 µg/mL | 0.71 | 0.29 | 1.77 | 0.467 |
| >85 µg/mL | 0.71 | 0.30 | 1.68 | 0.441 |
| MD | 0.34 | 0.07 | 1.57 | 0.166 |
| *Plasmodium* smears | 0.77 | 0.06 | 9.35 | 0.831 |
| **eGFR ≥47 & eGFR <65 & Ab** |  |  |  |  |
| Age (y.o.) | 1.17 | 1.09 | 1.25 | <0.001 |
| Male | 0.28 | 0.08 | 0.87 | 0.031 |
| MBP | 1.01 | 0.98 | 1.04 | 0.544 |
| Tabacco | 0.78 | 0.09 | 7.03 | 0.829 |
| PWV | 3.73 | 0.82 | 16.91 | 0.087 |
| Lymphopenia | NA | - | - | - |
| Hypereosinophilia | 0.95 | 0.71 | 1.28 | 0.755 |
| *Loa* MFD (Ref. 0 mf/mL) |  |  |  |  |
| 1-19.999mf/mL | 2.18 | 0.62 | 7.61 | 0.224 |
| >20.000 mf/mL | 4.97 | 0.41 | 60.67 | 0.209 |
| Ascaris | 0.87 | 0.26 | 2.85 | 0.813 |
| Trichuris | 1.19 | 0.37 | 3.81 | 0.766 |
| IgG-Plasmodium (Ref. ≤52 µg/mL) |  |  |  |  |
| 53-68 µg/mL | 0.20 | 0.02 | 2.08 | 0.178 |
| 69-85 µg/mL | 0.22 | 0.02 | 2.37 | 0.214 |
| >85 µg/mL | 0.91 | 0.20 | 4.12 | 0.900 |
| MD | 0.82 | 0.10 | 6.82 | 0.855 |
| *Plasmodium* smears | NA | - | - | - |
| **eGFR <47 & no Ab** |  |  |  |  |
| Age (y.o.) | 1.29 | 1.19 | 1.38 | <0.001 |
| Male | 0.12 | 0.03 | 0.44 | 0.001 |
| MBP | 0.97 | 0.93 | 1.00 | 0.081 |
| Tabacco | 4.10 | 0.83 | 20.31 | 0.083 |
| PWV | 1.87 | 0.44 | 7.99 | 0.397 |
| Lymphopenia | NA | - | - | - |
| Hypereosinophilia | 0.80 | 0.59 | 1.09 | 0.153 |
| *Loa* MFD (Ref. 0 mf/mL) |  |  |  |  |
| 1-19.999mf/mL | 0.28 | 0.05 | 1.47 | 0.133 |
| >20.000 mf/mL | NA | - | - | - |
| Ascaris | 0.65 | 0.18 | 2.31 | 0.507 |
| Trichuris | 1.70 | 0.48 | 5.94 | 0.406 |
| IgG-Plasmodium (Ref. ≤52 µg/mL) |  |  |  |  |
| 53-68 µg/mL | 0.24 | 0.02 | 3.10 | 0.278 |
| 69-85 µg/mL | 0.93 | 0.13 | 6.52 | 0.944 |
| >85 µg/mL | 0.80 | 0.13 | 4.92 | 0.811 |
| MD | 2.68 | 0.26 | 28.04 | 0.410 |
| *Plasmodium* smears | NA | - | - | - |
| **eGFR <47 & Ab** |  |  |  |  |
| Age (y.o.) | 1.16 | 1.04 | 1.29 | 0.006 |
| Male | 0.31 | 0.04 | 2.21 | 0.242 |
| MBP | 1.00 | 0.95 | 1.06 | 0.883 |
| Tabacco | 2.89 | 0.23 | 36.17 | 0.410 |
| PWV | 3.72 | 0.36 | 38.33 | 0.269 |
| Lymphopenia | NA | - | - | - |
| Hypereosinophilia | 0.75 | 0.29 | 1.97 | 0.560 |
| *Loa* MFD (Ref. 0 mf/mL) |  |  |  |  |
| 1-19.999mf/mL | 0.56 | 0.06 | 5.98 | 0.628 |
| >20.000 mf/mL | 12.54 | 0.79 | 198.17 | 0.072 |
| Ascaris | 0.47 | 0.06 | 3.51 | 0.459 |
| Trichuris | 2.09 | 0.28 | 15.52 | 0.473 |
| IgG-Plasmodium (Ref. ≤52 µg/mL) |  |  |  |  |
| 53-68 µg/mL | NA | - | - | - |
| 69-85 µg/mL | NA | - | - | - |
| >85 µg/mL | NA | - | - | - |
| MD | NA | - | - | - |
| *Plasmodium* smears | NA | - | - | - |

Abbreviations: MAP: mean arterial pressure; PWV: pulse wave velocity; eGFR: estimated glomerular filtration rate; MFD: microfilarial density; ACR: albumin- to creatinine ratio; CKD-EPI: Chronic Kidney Disease - Epidemiology Collaboration; EKFC: European Kidney Function Consortium.

**Supplementary Table 4. Adjusted relative risk ratio of independently associated variables with severity categories of CKD in saturated multinomial model using EKFC with Dubois correction.**

| **Ref. eGFR ≥90 & no Ab** | **Adjusted RRR** | **Low 95%*CI*** | **High 95%*CI*** | ***P*** |
| --- | --- | --- | --- | --- |
| **eGFR ≥90 & Ab** |  |  |  |  |
| Age (y.o.) | 0.99 | 0.96 | 1.01 | 0.247 |
| Male | 0.32 | 0.15 | 0.67 | 0.003 |
| MBP | 1.00 | 0.98 | 1.03 | 0.752 |
| Tabacco | 1.52 | 0.67 | 3.44 | 0.316 |
| PWV | 2.85 | 0.28 | 28.92 | 0.377 |
| Lymphopenia | 4.00 | 0.88 | 18.04 | 0.071 |
| Hypereosinophilia | 1.08 | 0.96 | 1.21 | 0.195 |
| *Loa* MFD (Ref. 0 mf/mL) |  |  |  |  |
| 1-19.999mf/mL | 2.22 | 1.14 | 4.34 | 0.019 |
| >20.000 mf/mL | 9.86 | 2.81 | 34.53 | <0.001 |
| Ascaris | 0.85 | 0.49 | 1.47 | 0.563 |
| Trichuris | 1.01 | 0.59 | 1.74 | 0.969 |
| IgG-Plasmodium (Ref. ≤52 µg/mL) |  |  |  |  |
| 53-68 µg/mL | 0.64 | 0.25 | 1.57 | 0.325 |
| 69-85 µg/mL | 0.60 | 0.23 | 1.53 | 0.283 |
| >85 µg/mL | 0.81 | 0.33 | 2.01 | 0.655 |
| MD | 0.78 | 0.24 | 2.48 | 0.669 |
| *Plasmodium* smears | 0.96 | 0.11 | 8.48 | 0.970 |
| **eGFR ≥65 & eGFR <90 & no Ab** | |  |  |  |
| Age (y.o.) | 1.13 | 1.11 | 1.15 | <0.001 |
| Male | 0.29 | 0.19 | 0.44 | <0.001 |
| MBP | 0.99 | 0.98 | 1.01 | 0.369 |
| Tabacco | 1.49 | 0.92 | 2.41 | 0.107 |
| PWV | 2.72 | 0.95 | 7.76 | 0.061 |
| Lymphopenia | 0.45 | 0.12 | 1.65 | 0.228 |
| Hypereosinophilia | 0.92 | 0.85 | 1.00 | 0.050 |
| *Loa* MFD (Ref. 0 mf/mL) |  |  |  |  |
| 1-19.999mf/mL | 0.93 | 0.63 | 1.36 | 0.704 |
| >20.000 mf/mL | 1.25 | 0.42 | 3.74 | 0.695 |
| Ascaris | 1.03 | 0.78 | 1.36 | 0.838 |
| Trichuris | 0.98 | 0.75 | 1.30 | 0.906 |
| IgG-Plasmodium (Ref. ≤52 µg/mL) |  |  |  |  |
| 53-68 µg/mL | 0.93 | 0.56 | 1.57 | 0.794 |
| 69-85 µg/mL | 1.19 | 0.70 | 2.03 | 0.522 |
| >85 µg/mL | 0.76 | 0.45 | 1.29 | 0.313 |
| MD | 0.44 | 0.20 | 1.00 | 0.049 |
| *Plasmodium* smears | 0.46 | 0.09 | 2.38 | 0.355 |
| **eGFR ≥65 & eGFR <90 & Ab** | |  |  |  |
| Age (y.o.) | 1.19 | 1.14 | 1.24 | <0.001 |
| Male | 0.10 | 0.04 | 0.22 | <0.001 |
| MBP | 1.00 | 0.97 | 1.02 | 0.696 |
| Tabacco | 1.80 | 0.59 | 5.51 | 0.300 |
| PWV | 1.68 | 0.41 | 6.88 | 0.472 |
| Lymphopenia | 0.47 | 0.04 | 5.44 | 0.544 |
| Hypereosinophilia | 0.84 | 0.67 | 1.05 | 0.115 |
| *Loa* MFD (Ref. 0 mf/mL) |  |  |  |  |
| 1-19.999mf/mL | 2.58 | 1.20 | 5.56 | 0.016 |
| >20.000 mf/mL | 13.35 | 3.09 | 57.69 | 0.001 |
| Ascaris | 0.85 | 0.43 | 1.68 | 0.636 |
| Trichuris | 1.22 | 0.62 | 2.40 | 0.555 |
| IgG-Plasmodium (Ref. ≤52 µg/mL) |  |  |  |  |
| 53-68 µg/mL | 1.03 | 0.33 | 3.18 | 0.962 |
| 69-85 µg/mL | 1.12 | 0.35 | 3.56 | 0.843 |
| >85 µg/mL | 0.93 | 0.31 | 2.80 | 0.889 |
| MD | 1.58 | 0.34 | 6.53 | 0.601 |
| *Plasmodium* smears | 0.64 | 0.05 | 8.54 | 0.735 |
| **eGFR ≥47 & eGFR <65 & no Ab** | |  |  |  |
| Age (y.o.) | 1.27 | 1.22 | 1.33 | <0.001 |
| Male | 0.10 | 0.05 | 0.19 | <0.001 |
| MBP | 1.00 | 0.98 | 1.01 | 0.631 |
| Tabacco | 4.64 | 1.85 | 11.64 | 0.001 |
| PWV | 2.54 | 0.75 | 8.55 | 0.132 |
| Lymphopenia | 0.22 | 0.02 | 2.39 | 0.215 |
| Hypereosinophilia | 0.92 | 0.78 | 1.07 | 0.272 |
| *Loa* MFD (Ref. 0 mf/mL) |  |  |  |  |
| 1-19.999mf/mL | 0.53 | 0.32 | 1.06 | 0.108 |
| >20.000 mf/mL | 1.78 | 0.29 | 10.95 | 0.535 |
| Ascaris | 0.95 | 0.52 | 1.72 | 0.854 |
| Trichuris | 1.04 | 0.58 | 1.87 | 0.901 |
| IgG-Plasmodium (Ref. ≤52 µg/mL) |  |  |  |  |
| 53-68 µg/mL | 0.95 | 0.38 | 2.37 | 0.921 |
| 69-85 µg/mL | 0.39 | 0.14 | 1.13 | 0.082 |
| >85 µg/mL | 0.62 | 0.25 | 1.55 | 0.303 |
| MD | 0.25 | 0.05 | 1.28 | 0.097 |
| *Plasmodium* smears | 1.62 | 0.18 | 14.50 | 0.668 |
| **eGFR ≥47 & eGFR <65 & Ab** |  |  |  |  |
| Age (y.o.) | 1.18 | 1.10 | 1.26 | <0.001 |
| Male | 0.24 | 0.08 | 0.81 | 0.021 |
| MBP | 1.00 | 0.97 | 1.04 | 0.811 |
| Tabacco | 1.89 | 0.35 | 10.15 | 0.457 |
| PWV | 7.46 | 1.48 | 37.58 | 0.015 |
| Lymphopenia | NA | - | - | - |
| Hypereosinophilia | 0.92 | 0.69 | 1.24 | 0.599 |
| *Loa* MFD (Ref. 0 mf/mL) |  |  |  |  |
| 1-19.999mf/mL | 1.62 | 0.48 | 5.39 | 0.435 |
| >20.000 mf/mL | 4.66 | 0.40 | 54.45 | 0.220 |
| Ascaris | 0.88 | 0.27 | 2.78 | 0.824 |
| Trichuris | 1.20 | 0.39 | 3.72 | 0.750 |
| IgG-Plasmodium (Ref. ≤52 µg/mL) |  |  |  |  |
| 53-68 µg/mL | 0.44 | 0.07 | 2.86 | 0.388 |
| 69-85 µg/mL | 0.25 | 0.02 | 2.59 | 0.243 |
| >85 µg/mL | 0.88 | 0.19 | 3.99 | 0.867 |
| MD | 0.75 | 0.09 | 6.15 | 0.786 |
| *Plasmodium* smears | NA | - | - | - |
| **eGFR <47 & no Ab** |  |  |  |  |
| Age (y.o.) | 1.35 | 1.24 | 1.47 | <0.001 |
| Male | 0.12 | 0.03 | 0.48 | 0.003 |
| MBP | 0.96 | 0.93 | 1.00 | 0.053 |
| Tabacco | 6.12 | 1.12 | 33.51 | 0.037 |
| PWV | 3.76 | 0.69 | 20.57 | 0.126 |
| Lymphopenia | NA | - | - | - |
| Hypereosinophilia | 0.86 | 0.63 | 1.19 | 0.375 |
| *Loa* MFD (Ref. 0 mf/mL) |  |  |  |  |
| 1-19.999mf/mL | 0.31 | 0.05 | 1.64 | 0.160 |
| >20.000 mf/mL | NA | - | - | - |
| Ascaris | 0.67 | 0.18 | 2.49 | 0.545 |
| Trichuris | 1.60 | 0.43 | 5.88 | 0.481 |
| IgG-Plasmodium (Ref. ≤52 µg/mL) |  |  |  |  |
| 53-68 µg/mL | 0.29 | 0.02 | 3.78 | 0.346 |
| 69-85 µg/mL | 0.47 | 0.05 | 4.04 | 0.488 |
| >85 µg/mL | 0.68 | 0.11 | 4.27 | 0.679 |
| MD | 0.65 | 0.03 | 12.34 | 0.777 |
| *Plasmodium* smears | NA | - | - | - |
| **eGFR <47 & Ab** |  |  |  |  |
| Age (y.o.) | 1.17 | 1.03 | 1.32 | 0.019 |
| Male | 0.20 | 0.02 | 2.00 | 0.172 |
| MBP | 1.00 | 0.95 | 1.06 | 0.885 |
| Tabacco | NA | - | - | - |
| PWV | 10.01 | 0.49 | 205.76 | 0.135 |
| Lymphopenia | NA | - | - | - |
| Hypereosinophilia | 0.86 | 0.38 | 1.94 | 0.717 |
| *Loa* MFD (Ref. 0 mf/mL) |  |  |  |  |
| 1-19.999mf/mL | 1.16 | 0.08 | 17.43 | 0.914 |
| >20.000 mf/mL | 49.99 | 1.63 | 1533.89 | 0.025 |
| Ascaris | NA | - | - | - |
| Trichuris | NA | - | - | - |
| IgG-Plasmodium (Ref. ≤52 µg/mL) |  |  |  |  |
| 53-68 µg/mL | NA | - | - | - |
| 69-85 µg/mL | NA | - | - | - |
| >85 µg/mL | NA | - | - | - |
| MD | NA | - | - | - |
| *Plasmodium* smears | NA | - | - | - |

Abbreviations: MAP: mean arterial pressure; PWV: pulse wave velocity; eGFR: estimated glomerular filtration rate; MFD: microfilarial density; ACR: albumin- to creatinine ratio; CKD-EPI: Chronic Kidney Disease - Epidemiology Collaboration; EKFC: European Kidney Function Consortium.

**Supplementary Table 5. Attributable fraction population and 95% confidence interval for having CKD in KDIGO classification using EKFC formula.**

|  | CKD ≥1 | CKD ≥1 with RAb |
| --- | --- | --- |
| Tobacco use | 3.5% [0.0–8.9] | 2.8% [0.0–10.4] |
| Arterial stiffness | 4.4% [0.0–10.1] | 3.7% [0.0–10.2] |
| Microfilaremic individuals | 14.7% [4.3–24.0] | 30.1% [16.2–42.8] |
| Female sex | 22.3% [10.2–32.7] | 23.2% [7.9–35.9] |

Abbreviations: RAb: renal abnormalities; CKD: chronic kidney disease; EKFC: European Kidney Function Consortium; KDIGO: Kidney Disease Improving Global Outcome

**Supplementary Figure 1. Probabilities of having renal abnormalities without CKD, or of having low grade CKD with renal abnormalities or higher grade CKD with renal abnormalities in those aged ≤50 vs >50 years.**

Dashed line: age ≤50 years, dotted line: age >50 years.
